# Improved performance of Cr(vi)-reducing microbial fuel cells by nano-FeS hybridized biocathodes†

**DOI:** 10.1039/d3ra00683b

**Published:** 2023-02-27

**Authors:** Xinglei Zhuang, Shien Tang, Weiliang Dong, Fengxue Xin, Honghua Jia, Xiayuan Wu

**Affiliations:** a College of Biotechnology and Pharmaceutical Engineering, Nanjing Tech University Nanjing 211816 China wuxiayuan@njtech.edu.cn +86 25 58139929 +86 25 58139929

## Abstract

Biocathode microbial fuel cells (MFCs) show promise for Cr(vi)-contaminated wastewater treatment. However, biocathode deactivation and passivation caused by highly toxic Cr(vi) and nonconductive Cr(iii) deposition limit the development of this technology. A nano-FeS hybridized electrode biofilm was fabricated by simultaneously feeding Fe and S sources into the MFC anode. This bioanode was then reversed as the biocathode to treat Cr(vi)-containing wastewater in a MFC. The MFC obtained the highest power density (40.75 ± 0.73 mW m^−2^) and Cr(vi) removal rate (3.99 ± 0.08 mg L^−1^ h^−1^), which were 1.31 and 2.00 times those of the control, respectively. The MFC also maintained high stability for Cr(vi) removal in three consecutive cycles. These improvements were due to synergistic effects of nano-FeS with excellent properties and microorganisms in the biocathode. The mechanisms were: (1) the accelerated electron transfer mediated by nano-FeS ‘electron bridges’ strengthened bioelectrochemical reactions, firstly realizing deep reduction of Cr(vi) to Cr(0) and thus effectively alleviating cathode passivation; (2) nano-FeS as ‘armor’ layers improved cellular viability and extracellular polymeric substance secretion; (3) the biofilm selectively enriched a diversity of bifunctional bacteria for electrochemical activity and Cr(vi) removal. This study provides a new strategy to obtain electrode biofilms for sustainable treatment of heavy metal wastewater.

## Abstract 2

Synergistic actions of nano-FeS coupling with biofilms realized deep reduction of Cr^6+^ to Cr^0^ and cathode passivation alleviation in MFCs.

## Graphical abstract

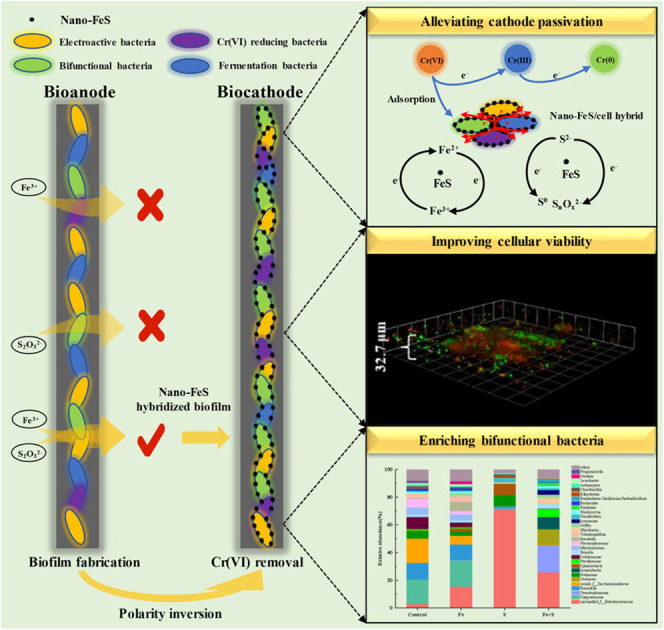

## 1. Introduction

Chromium poses a serious threat to the environment and human health. It is widely used in mining, smelting, leather tanning, and pigment manufacturing.^1,2^ Chromium in the environment is present primarily as Cr(vi) and Cr(iii). Cr(vi) is carcinogenic, teratogenic, mutagenic, and nonbiodegradable.^3^ It is characterized by extreme permeability and mobility.^4^ Cr(vi)-contaminated wastewater is typically treated by reducing toxic Cr(vi) to the less toxic Cr(iii) and then removing the latter by the precipitation of Cr(OH)_3_.^5^

Recently, bioelectrochemical systems (BESs), including microbial fuel cells (MFCs) and microbial electrolysis cells (MECs), have been used for removing heavy metals, recovering valuable metals, and generating electrical energy.^6^ Wang *et al.*^7^ first successfully removed 100 mg L^−1^ Cr(vi) at pH = 2 using MFCs with the chemical cathode. Tandukar *et al.*^8^ first demonstrated that mixed culture biocathodes exhibit superior Cr(vi) reduction efficiency over chemical cathodes in MFCs under neutral conditions. The biocathodes have attracted considerable attention owing to their regenerative capacity, mild reaction conditions, and high catalytic activity.^9^ Various means for improving Cr(vi) removal efficiency by biocathode MFCs have been reported. Yu *et al.*^10^ used polystyrene sulfonic acid and amino carbon nanotubes (NH_2_-CNT) to modify carbon cloth cathode in sediment microbial fuel cells (SMFCs) using layer-by-layer self-assembly, enhancing the Cr(vi) adsorption and bacterial attachment of the cathode; a variety of Cr(vi)-reducing bacteria were also selectively enriched, leading to a high (2.06 times higher than the control) Cr(vi) reduction rate. Zhao *et al.*^11^ used screened *Corynebacterium vitaeruminis* LZU47-1 to construct the biocathode, which yielded 53.4% and 52.32% higher power output and Cr(vi) removal efficiency, respectively, over a chemical cathode control. The introduction of exogenous adenylate cyclase-encoding gene in *Shewanella oneidensis* MR-1 enhanced the level of intracellular cAMP and thus enhanced bidirectional extracellular electron transfer (EET); the Cr(vi) reduction efficiency by the engineered strain (MR-1/pbPAC) was thus three times higher than that of the control.^12^ However, two bottlenecks remain in the development of biocathode MFCs for Cr(vi) removal in wastewater. First, the cathode EET remains low, especially with Cr(vi) reduction that produces cathode passivation *via* nonconductive Cr(iii) deposition.^13,14^ Second, cathodic microbial activity remains low, especially after exposure to high concentrations of Cr(vi) that releases toxic attack.^3,15^

Nano-FeS is an environmentally friendly iron-based material with high specific surface area, high reactivity, high electrical conductivity, and good biocompatibility.^16–18^ Nano-FeS can effectively reduce Cr(vi), promoted by Fe(ii) and S(ii).^16^ In addition, a high specific surface area provides more attachment sites for microorganisms and removes Cr(vi) by adsorption.^3,16,19^ Therefore, an increasing number of studies applied nano-FeS for Cr(vi) removal. For example, Ali *et al.*^20^ used FeS@rGO nanocomposites to modify carbon felt cathode in MFCs, affording a 4.6-fold increase in Cr(vi) removal; this improvement was attributed to conductivity and catalytic properties of the composites. Biogenic nano-FeS, compared with chemical nano-FeS, possesses superior properties and is green, inexpensive and readily available, presenting great promise for heavy metal removal.^21^ Synthesis of nano-FeS by *S. oneidensis* MR-1 and self-assembly to form nano-FeS/cell hybrids effectively enhanced electron transfer and microbial activity, resulting in a nearly five-fold increase of Cr(vi) removal.^21^ Qian *et al.*^22^ used sulfate-reducing bacteria (SRB) to synthesize nano-FeS to construct an enhanced electron transfer system (bio-FeS NP@SRB), which improved the kinetic constant of Cr(vi) removal 10-fold. Our previous work demonstrated that *in situ* synthesis of nano-FeS and self-assembly of a three-dimensional (3D) nano-FeS hybridized electrode biofilm could be successfully achieved by simultaneous feeding Fe and S sources into the mixed culture anode of a MFC, leading to considerably improved electron transfer and microbial activity. Our another previous work established a facile *ex situ* acclimation method of Cr(vi)-reducing biocathodes through polarity inversion of mature bioanodes to function as biocathodes.^23^ Therefore, it is logical to suspect the nano-FeS hybridized bioanode reversed as the Cr(vi)-reducing biocathode would improve cathode EET and microbial activity in MFCs, causing a high efficiency for Cr(vi)-contaminated wastewater treatment.

In order to validate the above hypothesis, this study introduced a nano-FeS hybridized electrode biofilm prepared in the anode to improve efficiency and service life of the Cr(vi)-reducing biocathode in a MFC. The acquisition of the nano-FeS hybridized electrode biofilm was initially fabricated by simultaneous feeding Fe and S sources into the mixed culture anode of a MFC, then reversing the hybridized bioanode as the biocathode to treat artificial Cr(vi)-containing wastewater. At the meantime, electrode biofilms prepared by separate feeding Fe or S source at the anode were conducted to compare effectiveness. The electrochemical performance and Cr(vi) removal of MFCs with different biocathodes were monitored. The changes in surface morphology, elemental valence, extracellular polymeric substances (EPS) secretion, and cellular viability of the electrode biofilms before and after Cr(vi) removal were comprehensively analyzed. In combination with the microbial community analysis, the impact mechanisms of the nano-FeS hybridized electrode biofilm for Cr(vi) removal in MFCs were finally elucidated.

## 2. Materials and methods

### 2.1. Anolyte and catholyte

Ultrapure water was used to prepare all the solutions. The anolyte was simulated wastewater with COD = 1000 mg L^−1^ (0.31 g L^−1^ NH_4_Cl, 2.452 g L^−1^ NaH_2_PO_4_·H_2_O, 4.576 g L^−1^ Na_2_HPO_4_, 0.13 g L^−1^ KCl, 1 g L^−1^ C_6_H_12_O_6_·H_2_O; pH = 7). The catholyte for the chemical cathode was phosphate buffer (2.452 g L^−1^ NaH_2_PO_4_·H_2_O, 4.576 g L^−1^ Na_2_HPO_4_, 0.13 g L^−1^ KCl; pH = 7) containing 40 mM potassium ferricyanide. The catholyte for the Cr(vi)-reducing biocathode was artificial Cr(vi) (40 mg L^−1^)-contaminated wastewater (0.28 g L^−1^ NH_4_Cl, 2.132 g L^−1^ NaH_2_PO_4_, 4.576 g L^−1^ Na_2_HPO_4_, 0.78 g L^−1^ KCl, 0.2 g L^−1^ NaHCO_3_, and 0.113 g L^−1^ K_2_Cr_2_O_7_; pH = 7).

### 2.2. Nano-FeS hybridized electrode biofilm fabrication

A dual-chamber MFC was constructed from cubic plexiglass chambers with effective volumes of 70 mL. Chambers were kept gastight and separated *via* a proton-exchange membrane (Nafion117, Dupont Co., USA),^24^ which was pretreated as described by Kim *et al.*^25^ The anode and cathode were carbon felts (4 cm × 4 cm × 0.5 cm), connected by 1 mm diameter titanium wires with a 1000 Ω external resistance. Prior to use, the carbon felts were soaked for 12 h each with 1 M NaOH and 1 M HCl sequentially, and then washed with deionized water until the pH was neutral. Anaerobic digester sludge from the Qiaobei Wastewater Treatment Plant (Nanjing, China) was the anodic inoculum, and the inoculation ratio was 1 : 2 (sludge : anolyte). The MFC was run in batch mode at 30 °C in a thermostatic biochemical incubator. The anolyte and catholyte were replaced every 3 d. The bioanode was considered to be mature after two consecutive cycles of stable voltage output of the MFC. The acclimation time of the bioanode was 18 days. Subsequently, the anolyte and catholyte were replaced to fabricate the nano-FeS hybridized electrode biofilm. Four MFC experimental groups were set as follows: (1) Fe + S: Fe source (5 mM FeCl_3_) and S source (5 mM Na_2_S_2_O_3_) were simultaneously added to the anolyte; (2) Fe: only Fe source (5 mM FeCl_3_) was added to the anolyte; (3) S: only S source (5 mM Na_2_S_2_O_3_) was added to the anolyte; (4) control: the anolyte was used without adding Fe and S sources. All the MFC groups were operated in the dark at 30 °C for eight cycles, with each cycle lasting 4 days. The chemical cathodes were applied for these biofilm fabrication MFCs. Operating conditions were identical except for the anolytes used for replacement in each cycle.

### 2.3. Cr(vi) removal experiment

The fabricated electrode biofilms were removed from the MFC anode chambers in an anaerobic incubator, gently rinsed with deoxygenated, deionized water, and transferred to the MFC cathode chambers as biocathodes for the Cr(vi) removal experiment. Predomesticated and mature bioanodes were placed in the anode chambers of the four Cr(vi)-reducing MFC groups. These bioanodes had similar potentials. In the Cr(vi) removal experiment, the anolyte and catholyte for the Cr(vi)-reducing biocathode described above were used. The Cr(vi) removal experiment ran for three cycles, with a reaction time of 10 h per cycle. Other operating conditions were consistent with the conditions described above.

### 2.4. Characterization and measurement

Voltages were recorded at 10 min intervals with a data acquisition unit (Keithley 2700). An electrochemical workstation CHI660E (Shanghai Chenhua, China) was used to measure cyclic voltammetry (CV) curves and electrochemical impedance spectra (EIS) in a three-electrode system.^26^ The cathode was the working electrode, Ag/AgCl was used as the reference electrode, and the anode was used as the counter electrode. CV was measured in a range from −0.80 to 0.80 V with a scan rate of 10 mV s^−1^; EIS frequency was set to a range from 100 kHz to 5 MHz with a potential amplitude of 10 mV.

MFC power density and polarization curves were obtained *via* linear sweep voltammetry (LSV) in a two-electrode system: the anode as the working electrode, the cathode and reference electrodes were the counter electrode, with a negative open circuit voltage as the starting point and a termination voltage of 0. The scanning rate was 1 mV s^−1^.^27^

A scanning electron microscope (SEM, JSM-5900, Japan) was used to image the surface of electrode biofilms.^27^ X-ray photoelectron spectroscopy (XPS, AXIS Ultra DLD, Shimadzu, UK) was used to assess the valence changes of Fe, S, and Cr on the electrode biofilms. The biocathode catholyte was sampled at 0, 0.5, 1, 2, 4, 6, 8, and 10 h for Cr(vi) detection. Cr(vi) content was assessed using diphenylcarbazide spectrophotometry.

EPS in electrode biofilms were primarily proteins (PN) and polysaccharides (PS), measured using a BCA Protein Assay Kit (P0010, Beyontian Biotechnology Co., Ltd, China) and the sulfuric acid–phenol method, respectively.^28^ A confocal laser scanning microscope (LSM880 with Airyscan, ZEISS, Germany) was used to assess cellular viability of electrode biofilms.^27^

### 2.5. High-throughput sequencing of electrode biofilms

A PowerSoil DNA kit (MoBio Laboratories Inc., USA) was used to extract DNA from biofilm samples. Samples were sent to Majorbio Bio-Pharm Technology Co. Ltd (Shanghai, China) for high-throughput sequencing of 16S rRNA genes using the Illumina MiSeq platform (Illumina, San Diego, USA). Amplification of the V3–V4 region of the 16S rRNA gene used primers 338F (5′-ACTCCTACGGGAGGCAGCAG-3′) and 806R (5′-GGACTACHVGGGTWTCTAAT-3′).^27^

## 3. Results and discussion

### 3.1. Electrochemical characteristics

**Fig. 1:**
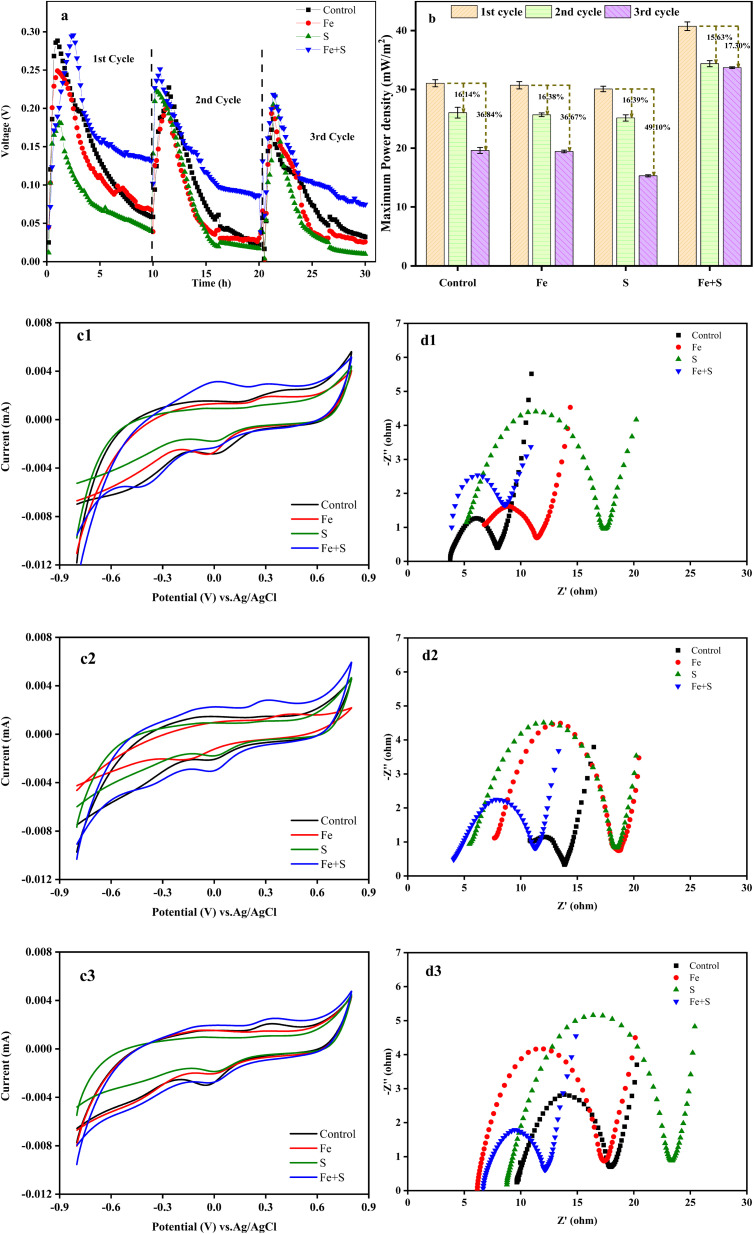
The voltage (a) and maximum power density (b) variations of different MFCs during the three Cr(vi) removal cycles; the cyclic voltammetry and electrochemical impedance spectroscopy (c1 and d1: 1st cycle; c2 and d2: 2nd cycle; c3 and d3: 3rd cycle) of different biocathodes.

Fig. 1a shows the variation of voltage outputs in the four MFC groups during three Cr(vi) removal cycles. Electricity generation from all the MFCs decreased considerably along with the operation of Cr(vi) removal cycles, indicating the occurrence of cathode passivation. The MFC with the Fe + S biocathode showed the highest voltage output and the slowest decline in each cycle, followed by the MFC with the control biocathode. Electricity generation from the MFCs with the Fe and S biocathodes decreased compared with the MFC with the control biocathode. Thus, the biocathode prepared with simultaneous feeding Fe and S sources promoted electricity production of the Cr(vi)-reducing MFC, but biocathodes prepared with feeding Fe or S source alone inhibited electricity production of the MFCs. Based on Fig. S1 and S2 (ESI†), nano-FeS particles (average size = 24.87 nm) were successfully synthesized at the MFC anode with simultaneous feeding Fe and S sources, and a nano-FeS hybridized electrode biofilm was fabricated. Therefore, the nano-FeS hybridized electrode biofilm as the Cr(vi)-reducing biocathode enhanced electricity generation of the MFC during Cr(vi) removal,^27^ while feeding Fe or S alone might produce nonconductive Fe salt precipitation or elemental S precipitation on the electrode biofilm that decreases electrochemical performance of the MFC.^29^

The maximum power density obtained from the MFC with the Fe + S biocathode in the first cycle was 40.75 ± 0.73 mW m^−2^ (Fig. 1b). This density decreased in the second and third cycles by 15.63% and 17.30%, respectively. These decreases were the least among all the MFCs. The maximum power density obtained from the MFC with the control biocathode in the second and third cycles decreased by 16.14% and 36.84%, respectively, from a high density of 31.05 ± 0.60 mW m^−2^ in the first cycle.

According to the CV curves (Fig. 1c1–c3), although the peak currents of the redox peaks and curve areas from all the MFCs decreased along with the operation of Cr(vi) removal cycles, the Fe + S biocathode had the largest peak current and curve area in each cycle. This demonstrated the prominent electrochemical activity of the Fe + S electrode biofilm. According to the EIS analysis (Fig. 1d1–d3), the ohmic resistance (*R*_s_) and charge-transfer resistance (*R*_ct_) of the control biocathode in the first cycle were 3.8 and 4.1 Ω, respectively. For the Fe + S biocathode, the *R*_s_ and *R*_ct_ in the first cycle were 3.7 and 4.6 Ω, respectively. Compared to the first cycle, the *R*_s_ and *R*_ct_ of the control biocathode in the third cycle increased by 153% and 105%, respectively, while the *R*_s_ and *R*_ct_ of the Fe + S biocathode in the third cycle increased by 65% and 17%, respectively. Overall, the internal resistance of all the biocathodes increased considerably after three cycles, and the Fe + S biocathode displayed the smallest increase. Consequently, the electrochemical analysis indicated that the cathode passivation due to the deposition of nonconductive Cr(vi) reduzates increased the internal resistance and decreased the electrochemical activity of the electrode biofilms, with a resulting decline in power generation of the MFCs;^30^ the Fe + S biocathode effectively alleviated the cathode passivation, avoiding the severe passivation observed in other experimental groups. The cathode passivation phenomenon has been commonly found in Cr(vi)-reducing MFCs.^8,30,31^ The subsequent XPS analysis (Fig. 4) of the electrode biofilms after Cr(vi) removal also confirmed the cathode passivation occurred in this study.

### 3.2. Cr(vi) removal

**Fig. 2:**
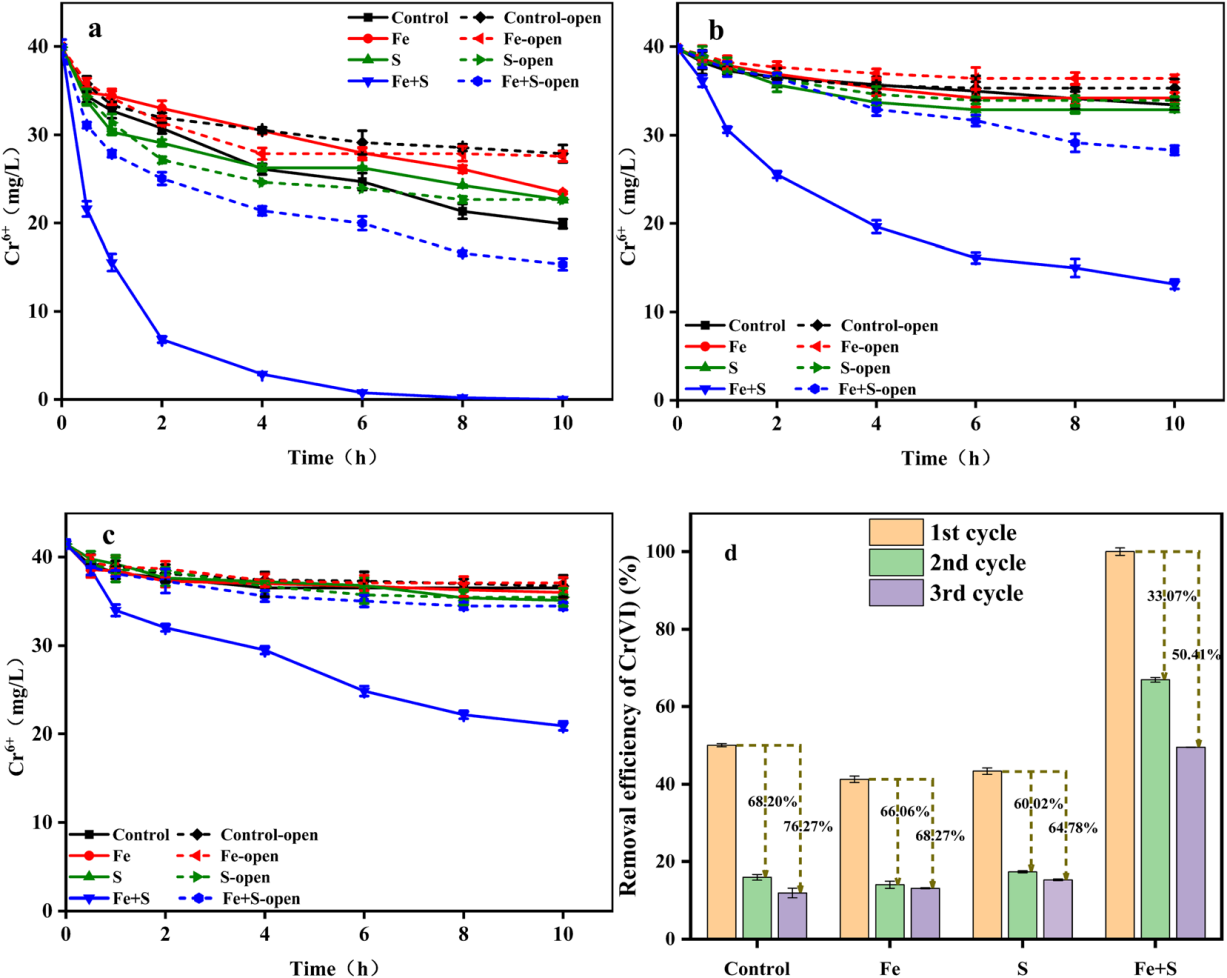
The changes of Cr(vi) concentration (a: 1st cycle; b: 2nd cycle; c: 3rd cycle) and Cr(vi) removal efficiency (d) in different MFCs.

Nano-FeS has been applied to remove Cr(vi) from water and soil, taking advantage of its ability to adsorb and reduce toxic Cr(vi).^16,32^ In order to clarify effects of nano-FeS and bioelectrochemistry, the Cr(vi) removal experiment was investigated under open and closed circuit conditions during the whole three cycles (Fig. 2a–c). The Cr(vi) removal ability in all the MFCs decreased to varying degrees along with the operation of three cycles, further confirming the occurrence of cathode passivation. The MFC with the Fe + S biocathode exhibited the highest Cr(vi) removal efficiency under both conditions in each cycle. Under open circuit condition, the MFC with the Fe + S biocathode achieved the highest Cr(vi) removal rate of 2.46 ± 0.02 mg L^−1^ h^−1^, followed by the MFC with the S biocathode (1.73 ± 0.07 mg L^−1^ h^−1^). The Cr(vi) removal rate was similar between the MFCs with the Fe biocathode (1.23 ± 0.03 mg L^−1^ h^−1^) and control biocathode (1.21 ± 0.01 mg L^−1^ h^−1^). After circuit connection, the Cr(vi) removal rate from the Fe + S group increased to 3.99 ± 0.08 mg L^−1^ h^−1^, twice the rate of the control group (2.00 ± 0.04 mg L^−1^ h^−1^). This implied that nano-FeS itself showed strong adsorption and reduction capacity for Cr(vi),^32^ and electrochemical processes further enhanced Cr(vi) removal in the nano-FeS hybridized electrode biofilm.^33^ Cr(vi) removal rates from the S and Fe groups were both lower than the rate from the control group after circuit connection. The reductive Fe and S salts generated during the biofilm fabrication process in the Fe and S electrode biofilms could improve Cr(vi) reduction under open circuit condition, while these nonconductive Fe and S salts might increase internal resistances of the electrode biofilms to inhibit Cr(vi) reduction under closed circuit condition. By the third cycle, except for the Fe + S group, the Cr(vi) removal rates from other three groups were basically the same under both the open and closed circuit conditions, denoting serious cathode passivation in these three electrode biofilms hindered electrochemical processes for Cr(vi) reduction.

Fig. 2d shows the Cr(vi) removal efficiency of different MFC groups under closed circuit condition across the three operating cycles. The highest Cr(vi) removal efficiency (100% ± 0.95%) was obtained from the Fe + S group in the first cycle, which decreased by 33.07% and 50.41% in the second and third cycles, respectively. The Cr(vi) removal efficiency from the control group in the first cycle was 50.11 ± 0.41%, which decreased by 68.20% and 76.27% in the second and third cycles, respectively. Thus, severe cathode passivation occurred in the control, Fe, and S groups, while the Fe + S electrode biofilm effectively alleviated cathode passivation, which was consistent with the above electrochemical performance. Song *et al.*^34^ fabricated a graphene hybridized electrode biofilm by injecting graphene oxide into the anode of a MFC; this electrode biofilm was then reversed as the biocathode to completely remove 40 mg L^−1^ Cr(vi) within 48 h in a MFC. Herein, the removal time for 40 mg L^−1^ Cr(vi) by the nano-FeS electrode biofilm was shortened to 10 h, which proved the superiority of the nano-FeS hybridized electrode biofilm for Cr(vi) removal in MFCs.

### 3.3. Characteristic analyses of electrode biofilms

#### 3.3.1. Morphology analysis

**Fig. 3:**
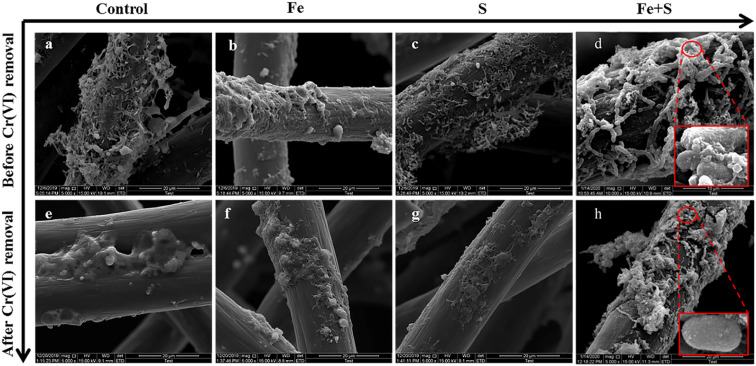
The SEM images of different electrode biofilms before (a–d) and after (e–h) Cr(vi) removal (the red boxes in (d) and (h) show the amplification versions of the circle parts).

**Fig. 4:**
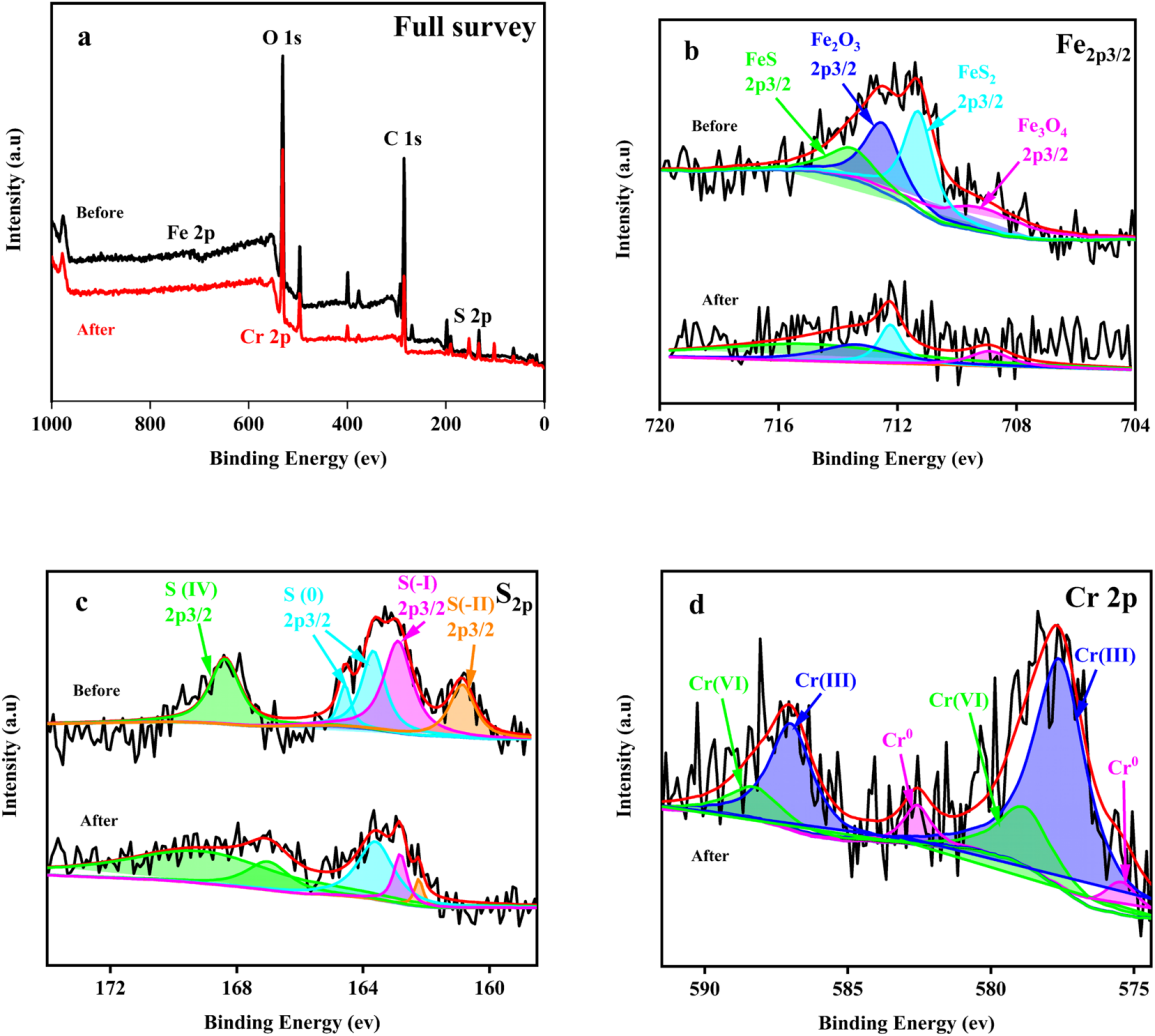
The full survey (a), Fe_2p_3/2__ (b), S_2p_ (c) and Cr_2p_3/2__ (d) XPS spectra of the Fe + S electrode biofilm before and after Cr(vi) removal.

SEM images of the four electrode biofilms before and after Cr(vi) removal showed considerable changes in surface morphology (Fig. 3). Before Cr(vi) removal, numerous rod-shaped bacteria were attached to all the electrodes. The control and Fe + S electrode biofilms displayed the largest bacterial populations. Many round particles were observed on the Fe + S electrode biofilm. These particles were not only attached to the microbial cell surface to form ‘armor’ layers but also as ‘electron bridges' to connect individual bacteria into a 3D network (Fig. 3d). Our previous work^27^ and XPS analysis (Fig. 4) afterwards both confirmed that these round particles were nano-FeS particles synthesized by the bioanode during the biofilm fabrication process. Jiang *et al.*^35^ also found that a nano-FeS/cell 3D network biofilm with excellent conductivity was constructed by nano-FeS bridging cells after *in situ* nano-FeS synthesis using *Shewanella* PV-4. The surface of the Fe electrode biofilm was covered with more deposits relative to other electrode biofilms. These deposits could be iron phosphate and other iron precipitates formed by the reactions of Fe(iii) with the phosphate buffer.

After Cr(vi) removal, the number of bacteria on all the electrodes considerably decreased, and many deposits and sticky substances encapsulated bacterial cells. Deposits observed after Cr(vi) removal might be products of Cr(vi) reduction. These viscous substances could be PS secreted by microbial cells after Cr(vi) toxic shock, which is a common stress response of microorganisms.^36^ The Fe + S electrode biofilm showed relatively fewer encapsulated bacteria and the largest number of intact bacteria; this was because the nano-FeS ‘armor’ layers could protect cells against toxic Cr(vi), resulting in less secretion of viscous PS and presence of more healthy and plump cells (Fig. 3h).

#### 3.3.2. Elemental composition analysis

XPS analysis was performed on the Fe + S electrode biofilm before and after Cr(vi) removal (Fig. 4). The presence of FeS on the electrode biofilm before Cr(vi) removal was detected, indicating successful synthesis and fabrication of nano-FeS on the biofilm (Fig. 4b and Table S1†). Some oxidation products of FeS, such as FeS_2_, Fe_3_O_4_, and Fe_2_O_3_, were also detected on the biofilm, which might be due to the sample preparation process for XPS analysis.^27^ In general, the proportion of Fe(ii) on the biofilm before Cr(vi) removal was two-fold higher than that of Fe(iii), indicating considerable reduction capacity of the nano-FeS electrode biofilm. After Cr(vi) removal, the Fe(ii)/Fe(iii) ratio on the Fe + S electrode biofilm was close to 1, showing that Fe(ii) played an important role in Cr(vi) reduction.

The S_2p_ map before Cr(vi) removal (Fig. 4c) indicated that S(0) and S(iv) in S source (Na_2_S_2_O_3_) were reduced to S(i) and S(ii) by microorganisms during the biofilm fabrication process. After Cr(vi) removal, the proportion of S(iv) increased by 1.62 fold and the proportion of S(0), S(i) and S(ii) decreased to varying degrees (Table S2, ESI†). Thus, S(0), S(i), and S(ii) also played important roles in Cr(vi) reduction. S(ii) decreased the most (78.90%), reflecting the key role of nano-FeS in Cr(vi) reduction.

Cr(vi) was reduced to Cr(iii) and Cr(0), with a Cr(iii)/Cr(0) of 9.56 (Table S3, ESI†). Cr(vi) was only reduced to Cr(iii) on the control electrode biofilm(Fig. S3, ESI†), which is consistent with existing relevant studies.^26^ Deep reduction of Cr(vi) to Cr(0) was firstly realized in the biocathode MFC, which was likely attributed to the combined actions of nano-FeS and microorganisms of the nano-FeS hybridized electrode biofilm; this combination reduced the potential or activation energy of Cr(vi) reduction.^20,37^ In addition, the reductive environment of the MFC cathode promoted the rapid conversion of Fe(iii) to Fe(ii), which contributed to deep reduction of Cr(vi).^21,38^ The deposition of conductive Cr(0) on the electrode surface could alleviate cathode passivation to some extent, improving EET and thus Cr(vi) reduction.

#### 3.3.3. EPS secretion and microbial activity analysis

**Fig. 5:**
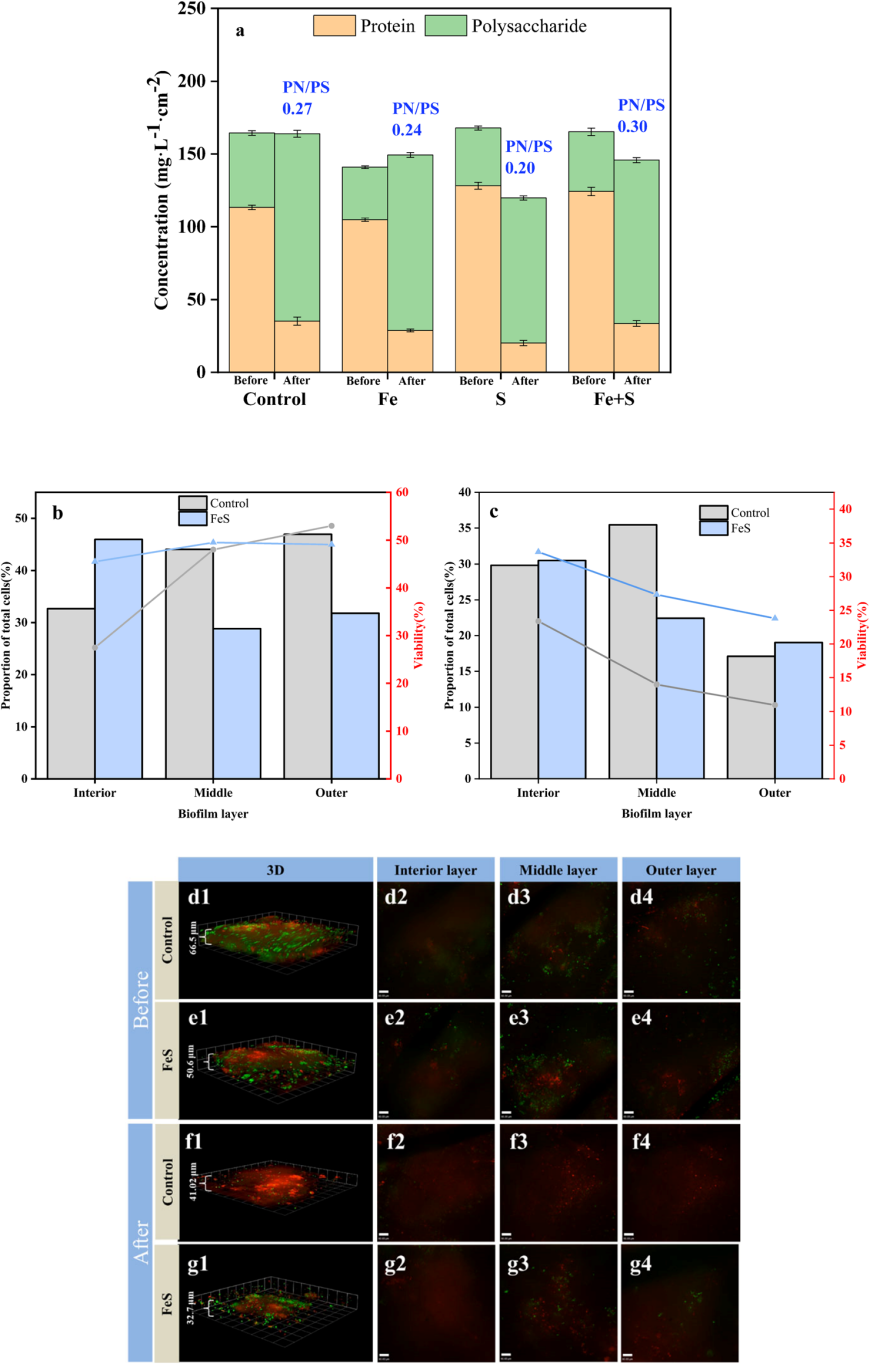
EPS concentrations (a) of different electrode biofilms before and after Cr(vi) removal; biomass and cellular viability (b: before; c: after) based on CLSM images (d and e: before; f and g: after) in different layers of the control and Fe + S electrode biofilms before and after Cr(vi) removal.

EPS in the biofilm matrix, mainly including PN and PS, can protect microorganisms from toxicity.^39^ EPS composition and content before Cr(vi) removal did not show notable differences among the four electrode biofilms, and PN/PS ratios of these biofilms were >2 (Fig. 5a). Some aromatic proteins in EPS can promote EET, and a high PN/PS ratio is conducive to efficient and stable electrode biofilm formation.^36,40,41^ Compared with the control electrode biofilm, the addition of Fe and S sources and the synthesis of nano-FeS during the biofilm fabrication process did not have a significant impact on microbial metabolism of the biofilm.

After Cr(vi) removal, all the electrode biofilms showed considerable decreases in PN concentrations and concomitant increases in PS concentrations. The PN/PS ratio of the Fe + S electrode biofilm was the largest (0.30), followed by the control electrode biofilm (0.27), and the Fe (0.24) and S (0.20) electrode biofilms had smaller PN/PS ratios. The results were consistent with the electrochemical results discussed above. Increased PS secretion is a response of microbial cells to stress in adverse environments, confirming the phenomenon observed by SEM mentioned above.^36^ However, PS is nonconductive. Large amounts of PS can negatively affect EET in biofilms, leading to declined performance of electrode biofilms.^11,42^ Therefore, the high PN/PS ratio of the Fe + S electrode biofilm was beneficial for continuous treatment of pollutants.

CLSM was used to analyze changes in microbial activity of the Fe + S and control electrode biofilms before and after Cr(vi) removal (Fig. 5b–g). Before Cr(vi) removal, CLSM images (Fig. 5d and e) showed little differences of biomass and cellular viability between these two electrode biofilms. The quantitative results of biomass and cellular viability in different biofilm layers (Fig. 5b) showed that biomass and cellular viability of the control electrode biofilm gradually increased from the inner to the outer layer. Biomass of the Fe + S electrode biofilm first decreased and then increased slightly from the inner to the outer layer, while cellular viability increased first and then decreased slightly, consistent with our previous work.^27^ Electron transfer resistance of a traditional electrode biofilm increased from the outer to the inner layer, leading to decreased biomass and cellular viability along the same direction.^43^ The Fe + S electrode biofilm was a conductive 3D network with the help of nano-FeS ‘electron bridges’; hence, the cellular viability had no notable differences among different biofilm layers; biomass in the middle and outer layers was slightly less compared with those in the control electrode biofilm, as nano-FeS particles occupied some space for bacterial growth.^27^

After Cr(vi) removal, CLSM images (Fig. 5f and g) showed considerably thinner biofilms, and nonviable cells increased considerably in the control electrode biofilm. In contrast, the proportion of living cells in the Fe + S electrode biofilm was considerably higher than that in the control electrode biofilm. Cr(vi) showed greater toxicity to bacterial cells in the control electrode biofilm, and the Fe + S electrode biofilm showed enhanced tolerance to Cr(vi) due to the presence of nano-FeS. The quantitative results (Fig. 5c) showed that biomass and cellular viability of these two electrode biofilms decreased considerably in each layer, especially for the control electrode biofilm. Biomass and cellular viability of these two electrode biofilms in the outer layer were the lowest, indicating that the outer biofilm layer suffered the most severe toxic attack. After Cr(vi) removal, biomass and cellular viability in the outer layer of the Fe + S electrode biofilm exceeded those of the control electrode biofilm by 0.11- and 1.17-fold, respectively. This confirmed that the nano-FeS ‘armor’ layers could protect microbial cells from toxic attack, conducive to continuous treatment of pollutants by electrode biofilms.

### 3.4. Microbial community analysis

**Fig. 6:**
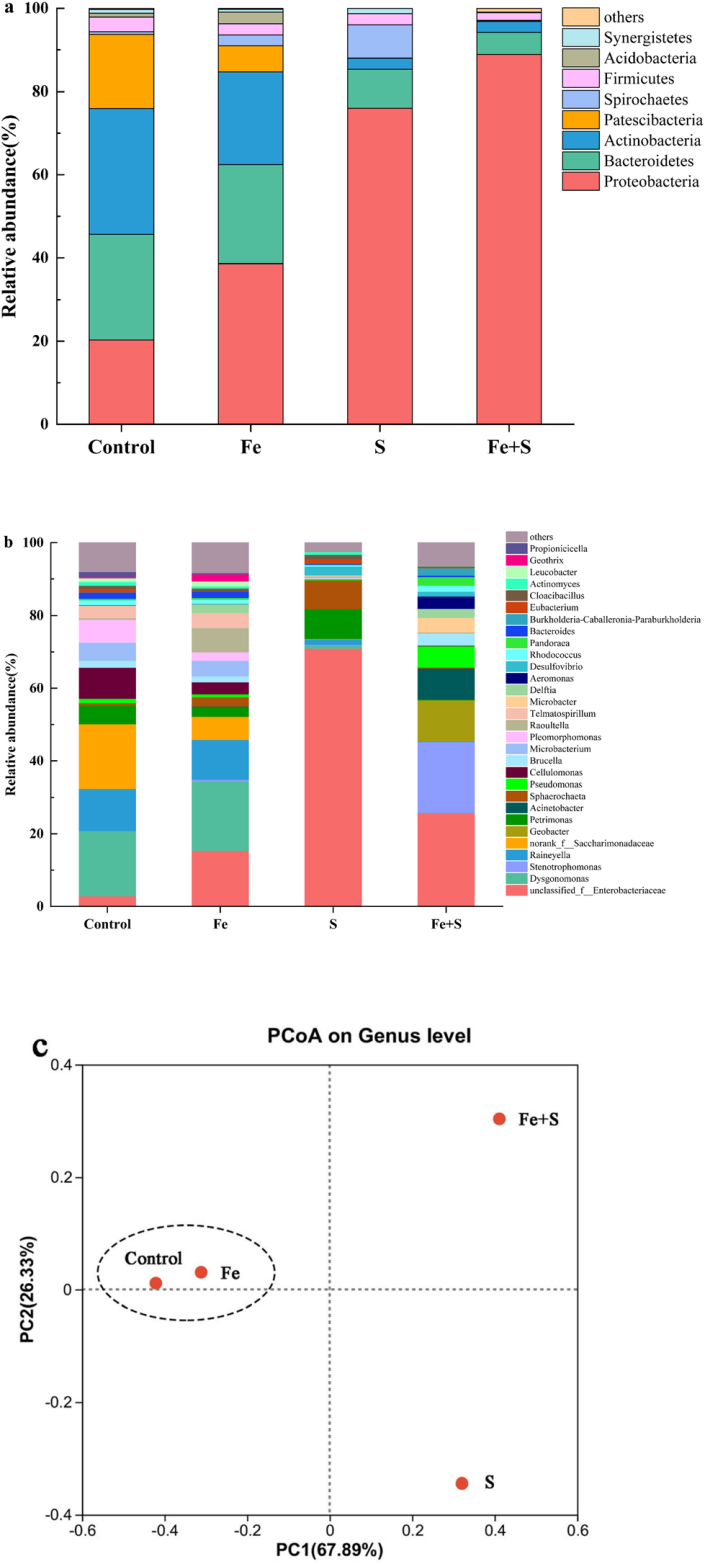
Microbial community compositions at the phylum (a) and genus (b) level, and principal coordinate analysis (c) based on the microbial community at the genus level of different electrode biofilms after Cr(vi) removal.

The Fe + S electrode biofilm after Cr(vi) removal showed the richest microbial diversity, followed by the control, and the Fe and S electrode biofilms exhibited lower diversity (Table S4, ESI†). Fig. 6 presents microbial community compositions of different electrode biofilms after Cr(vi) removal. At the phylum level (Fig. 6a), the control electrode biofilm had four dominant phyla: *Actinobacteria* (30.32%), *Bacteroidetes* (25.34%), Proteobacteria (20.31%), and *Patescibacteria* (17.74%). The Fe + S electrode biofilm was dominated by two phyla: Proteobacteria (88.89%) and *Bacteroidetes* (5.33%). The Fe electrode biofilm mirrored the dominant phylum types in the control electrode biofilm but relative abundance differed: Proteobacteria (38.67%), *Bacteroidetes* (23.78%), *Actinobacteria* (22.25%), and *Patescibacteria* (6.30%). The S electrode biofilm possessed three primary phyla: Proteobacteria (76.01%), *Bacteroidetes* (9.34%), and *Spirochaetes* (7.98%). Hence, both the Fe + S and S electrode biofilms were selectively enriched with Proteobacteria. Species in this phylum are frequently identified in MFCs and many are electroactive Cr(vi)-reducing strains.^44–46^*Actinobacteria* displays some Cr(vi) tolerance and reduction ability and is often found in MFC biocathodes used for heavy metal removal.^9^

At the genus level (Fig. 6b), the dominant genera in the control electrode biofilm were *Dysgonomonas* (17.85%), *Saccharimonadaceae* (17.72%), and *Raineyella* (11.66%). Dominant genera in the Fe + S electrode biofilm were *Enterobacteriaceae* (25.71%), *Stenotrophomonas* (19.35%), *Geobacter* (11.40%), and *Acinetobacter* (8.50%). Dominant genera in the Fe electrode biofilm were *Dysgonomonas* (18.94%), *Enterobacteriaceae* (15.38%), and *Raineyella* (10.99%). In the S electrode biofilm, dominant genera were mainly *Enterobacteriaceae* (70.96%). *Dysgonomonas* is thought to be involved in the reduction of Cr(vi) at BES biocathodes.^9,47^*Saccharimonadaceae* and *Raineyella* are also associated with Cr(vi) sorption and removal.^48,49^*Enterobacteriaceae*, belonging to the phylum of Proteobacteria, is a genus of electrochemically active bacteria, and our previous work demonstrated that these microbes were selectively enriched in the nano-FeS hybridized electrode biofilm.^27^*Stenotrophomonas*, another genus of electrochemically active bacteria in the phylum of Proteobacteria, was enriched in a BES biocathode for Cr(vi) removal.^50^*Geobacter* is a typical electroactive genus that effectively reduces many heavy metals, such as Cr(vi).^51^*Acinetobacter* is a genus of electroactive sulfide oxidizing bacteria with significant tolerance and reduction capacity for Cr(vi).^52–54^ Apparently, the Fe + S electrode biofilm selectively enriched a diversity of bifunctional bacteria with both electrochemical activity and Cr(vi) removal capacity. The presence of these microbes strengthened electricity production and Cr(vi) removal of biocathode MFCs.

A principal coordinate analysis at the genus level of different electrode biofilms after Cr(vi) removal (Fig. 6c) showed a distinct cluster for the Fe and control electrode biofilms away from the Fe + S and S electrode biofilms. Hence, the addition of Fe source alone had little impact on microbial community structure in the electrode biofilm; conversely, the addition of S source alone and Fe and S sources simultaneous considerably altered the microbial community structures; only simultaneous feeding Fe and S sources exerted a positive impact on microbial community structure in the electrode biofilm.

## 4. Conclusion

In this study, electrode biofilms were prepared by simultaneous or separate dosing of Fe and S sources to the anodes of MFCs. The effectiveness of reversing these electrode biofilms as MFC biocathodes for Cr(vi) removal was then investigated. The MFC with the Fe + S biocathode attained the maximum power output (40.75 ± 0.73 mW m^−2^) and Cr(vi) removal rate (3.99 ± 0.08 mg L^−1^ h^−1^), which were 1.31 and 2.00 times as high as those of the control, respectively. Nano-FeS enhanced electron transfer and bioelectrochemical reduction, realizing deep reduction of Cr(vi) to Cr(0) and alleviating cathode passivation. Moreover, nano-FeS formed ‘armor’ layers and ‘electron bridges’ on microbial cells, which improved biofilm activity, EPS secretions, and microbial community structure. The strategy of electrode biofilm fabrication increased the efficiency and service life of Cr(vi)-reducing biocathodes in MFCs, showing great application potential in heavy metal wastewater treatment.

## Author contributions

Xinglei Zhuang: investigation and writing original draft; Shien Tang: investigation and data curation; Weiliang Dong: software; Fengxue Xin: methodology; Honghua Jia: project administration; Xiayuan Wu: supervision and funding acquisition.

## Conflicts of interest

The authors declare no conflicts of interest to this work.

## Supplementary Material

RA-013-D3RA00683B-s001
