# Supplementary material for: Improved performance of Cr(vi)-reducing microbial fuel cells by nano-FeS hybridized biocathodes
Source: RSC Adv. 2023 Feb 27;13(10):6768–78. doi: 10.1039/d3ra00683b (PMC9969982; doi:10.1039/d3ra00683b)
Supplement: RA-013-D3RA00683B-s001 [file RA-013-D3RA00683B-s001.pdf]

Electronic Supplementary Material (ESI) for RSC Advances.
This journal is © The Royal Society of Chemistry 2023

Supporting Information

Improved performance of Cr(VI)-reducing microbial fuel cells by nano-FeS hybridized biocathodes

Xinglei Zhuang[1], Shien Tang[1], Weiliang Dong, Fengxue Xin, Honghua Jia, Xiayuan Wu*

College of Biotechnology and Pharmaceutical Engineering, Nanjing Tech University, Nanjing 211816, China

***Corresponding author:**

Xiayuan Wu

Nanjing Tech University, No.30 Puzhu Road(S), Nanjing, 211816, Jiangsu, P.R. China

Tel./Fax: +86 25 58139929

E-mail: wuxiayuan@njtech.edu.cn

[1]These authors contributed equally to the article.

FIGURES (3)

TABLES (4)

PAGES (8)

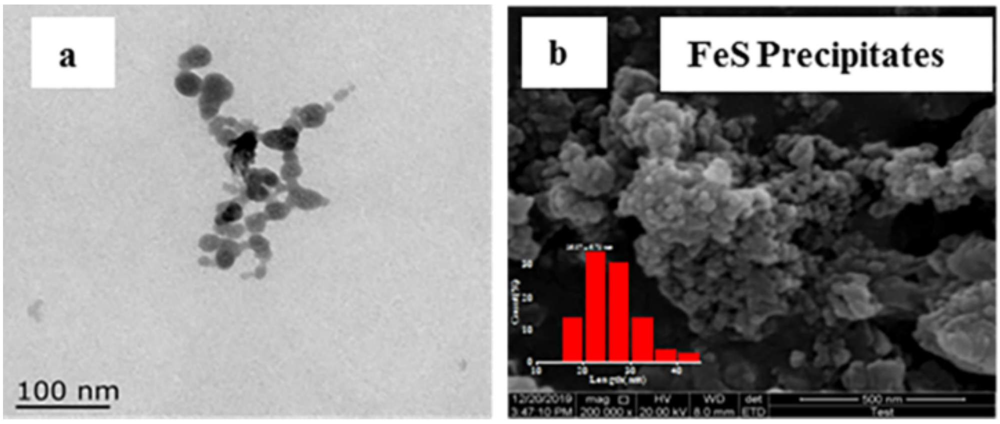

Fig. S1 The TEM (a) and SEM (b) images of produced precipitates in the Fe+S MFC for electrode biofilm fabrication.

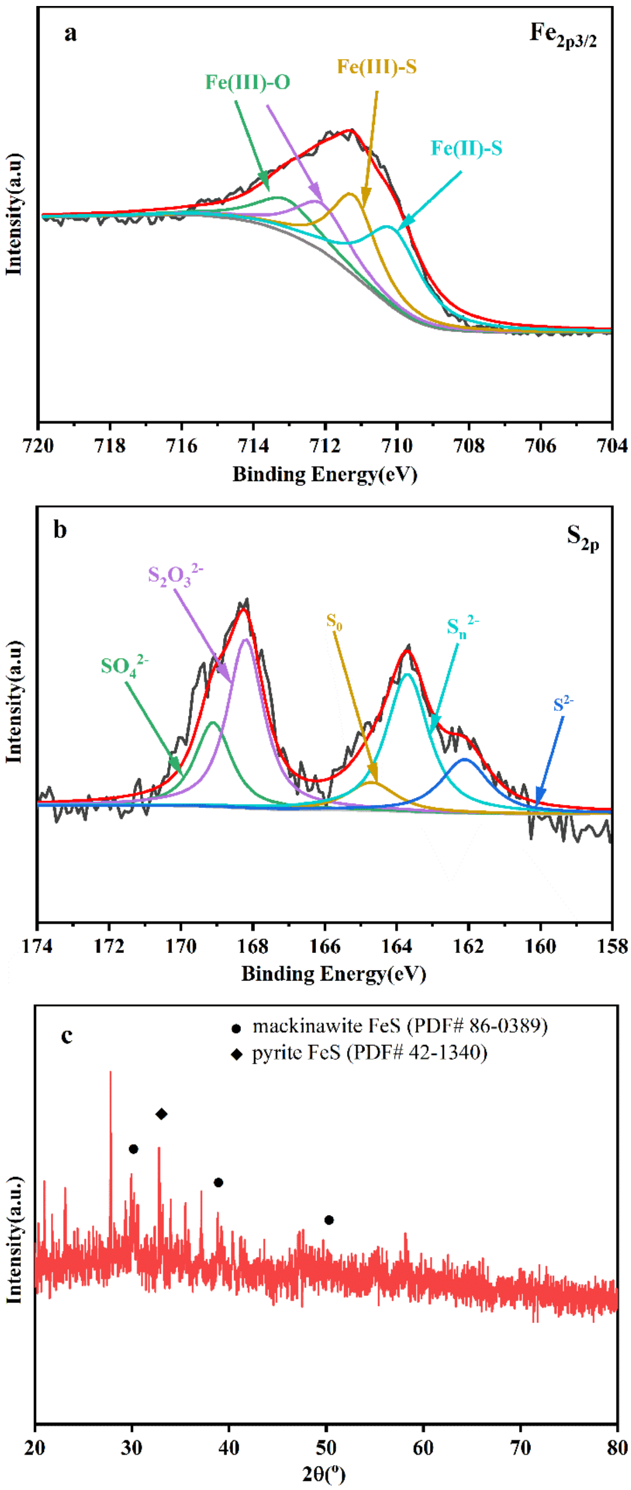

Fig. S2 The XPS spectra (a: $Fe_{2p3/2}$; b: S_2p) and XRD pattern (c) of produced precipitates in the Fe+S MFC for electrode biofilm fabrication.

Table S1 The distribution (Wt%) of different valence Fe compounds.

Group	FeS	Fe_2O_3	FeS_2	Fe_3O_4
Before Cr(VI) removal	32.53%	21.69%	42.91%	2.87%
After Cr(VI) removal	27.36%	35.07%	23.78%	13.79%

Table S2 Valence distribution (Wt%) of S element.

Group	S(IV)	S(0)	S(-I)	S(-II)
Before Cr(VI) removal	24.15%	32.15%	31.33%	12.37%
After Cr(VI) removal	63.16%	26.11%	8.12%	2.61%

Table S3 Valence distribution (Wt%) of Cr element.

Valence state of element	Wt(%)
Cr(VI)	23.03%
Cr(III)	69.68%
Cr(0)	7.29%

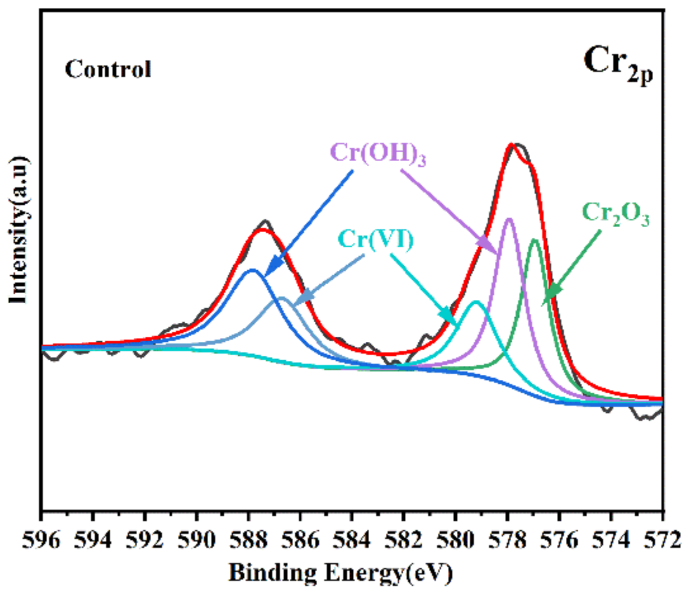

Fig. S3 The XPS spectra of the Control electrode biofilm after Cr(VI) removal.

Table S4 The α-diversity indices of microbial community in different electrode biofilms after Cr(VI) removal.

Group	OTU	ACE	Chao1	Shannon	Simpson
**Control**	254	404.1156	342.0286	3.0915	0.0802
**Fe**	212	256.1966	255.3846	3.2939	0.0625
**S**	171	225.6967	213.0000	1.7742	0.3478
**Fe+S**	275	288.3727	288.5938	2.9606	0.1015
